# An Algorithm for Online Stochastic Error Modeling of Inertial Sensors in Urban Cities

**DOI:** 10.3390/s23031257

**Published:** 2023-01-21

**Authors:** Luodi Zhao, Long Zhao

**Affiliations:** 1 School of Automation Science and Electrical Engineering, Beihang University, Beijing 100191, China; 2 Digital Navigation Center, Beihang University, Beijing 100191, China; 3 Science and Technology on Aircraft Control Laboratory, Beihang University, Beijing 100191, China

**Keywords:** GMWM, stochastic error, inertial sensor, sensor calibration, error model, Allan variance

## Abstract

Regardless of whether the global navigation satellite system (GNSS)/inertial navigation system (INS) is integrated or the INS operates independently during GNSS outages, the stochastic error of the inertial sensor has an important impact on the navigation performance. The structure of stochastic error in low-cost inertial sensors is quite complex; therefore, it is difficult to identify and separate errors in the spectral domain using classical stochastic error methods such as the Allan variance (AV) method and power spectral density (PSD) analysis. However, a recently proposed estimation, based on generalized wavelet moment estimation (GMWM), is applied to the stochastic error modeling of inertial sensors, giving significant advantages. Focusing on the online implementation of GMWM and its integration within a general navigation filter, this paper proposes an algorithm for online stochastic error calibration of inertial sensors in urban cities. We further develop the autonomous stochastic error model by constructing a complete stochastic error model and determining model ranking criterion. Then, a detecting module is designed to work together with the autonomous stochastic error model as feedback for the INS/GNSS integration. Finally, two experiments are conducted to compare the positioning performance of this algorithm with other classical methods. The results validate the capability of this algorithm to improve navigation accuracy and achieve the online realization of complex stochastic models.

## 1. Introduction

Modeling and estimation of inertial sensor errors are generally challenging tasks, especially for low-cost inertial micro-electromechanical system (MEMS) sensors, since the error model has complex spectral structures. For a global navigation satellite system (GNSS) and inertial navigation system (INS) integrated system, it is usually performed through a general Kalman filter, e.g., an extended Kalman filter (EKF), which is closely related to the inertial sensor modeling. When the GNSS signals are partially or completely unavailable, the INS operates in coasting mode, i.e., the navigation parameters can be estimated completely independently of the GNSS. Consequently, the overall navigation performance depends greatly on the accuracy of the inertial signal, or more precisely, on the errors of the inertial signal. These errors are integrated into the INS, and their impact increases dramatically over time. In conclusion, accurate modeling and estimation of the error of inertial signals are crucial for improving the quality of navigation performance.

The errors of inertial sensors can generally be divided into deterministic errors and stochastic errors. Most deterministic errors can be compensated for by physical models and have been widely studied [1,2,3], while stochastic errors are difficult to model. This is because there are many influence factors and normally the model is too complex to estimate correctly. Traditional estimation methods, such as Allan variance (AV) and power spectral density (PSD) analysis methods, have obvious disadvantages when the stochastic error structure is complex [4]. AV is currently the most widely used method in engineering to identify and calibrate inertial sensors [5,6,7,8,9]. Although this method was originally intended to study the stability of oscillators, it has been successfully applied to problems in a large number of different types of sensors, among which is the modeling of inertial sensor errors. However, AV is only suitable for stochastic processes that can be clearly identified and separated in the spectral domain. More strictly, stochastic processes are thought not to be affected by spectral ambiguity, while spectral ambiguity is common for low-cost MEMS inertial measurement units (IMU) [10,11]. Since human judgment is required in the identification of noise when performing Allan variance, it will absolutely cause deviation in estimation parameters in most cases. For PSD analysis, the periodogram is an inconsistent estimator of the power spectral density function and can be badly biased even for large sample sizes (because of frequency leakage effects). Moreover, when the PSD has large variability over a very narrow frequency band, it will make the least squares optimization problem based on the difference between the empirical PSD and the model-based PSD more difficult to solve [12]. However, reference [13] associated the wavelet variance (WV) with the PSD, and WV can be calculated from the samples using the wavelet transform estimator. Accordingly, reference [14] proposed a generalized wavelet moment estimation method, which identifies the time series to be estimated as a combination of stochastic processes. In such cases, the GMWM estimator is asymptotically consistent, and the empirical WV of the series corresponds to the WV implied by the assumed model. The generalized least squares method has been adopted to minimize the discrepancy between the two and to estimate the parameters of the latter [15]. This method can effectively avoid the disadvantages of the above traditional methods and has significant practical application value. In addition to the modeling of a given sequence of IMU data, other researchers have studied the modeling of IMU error within filters. Reference [16] introduced the stochastic IMU error models within a Sage Husa adaptive robust Kalman filter. Reference [17] developed an adaptive Kalman filter with colored noise for gyroscope random drift. However, these researchers calibrated inertial sensors before or after the experiments, i.e., offline calibration. Moreover, they hardly considered the time-taken for the calibration process. For these reasons, we propose an algorithm for online modeling of inertial sensor errors based on GMWM and mainly focus on the modeling of the stochastic error of the vehicle-mounted inertial sensors in urban areas.

The algorithm in this work is designed under the frame of INS/GNSS integration within KF. The main contributions of this paper are as follows: Firstly, this paper realizes autonomous stochastic error modeling by constructing a complete stochastic error model and a model ranking criterion. Secondly, this paper proposes a static state detecting algorithm with an adaptive threshold. It collects and accumulates static data when the vehicle stops. Finally, the frame of INS/GNSS integration is developed, combining autonomous stochastic error modeling and detecting module as feedback within the EKF. It realizes online modeling of the stochastic errors and provides a more accurate navigation solution as the vehicle runs. Two experiments, the GNSS denial experiment and GNSS available experiment, are designed to validate the feasibility of the proposed algorithm.

The rest of the paper is organized as follows: Section 2 introduces the main contributions of this paper. It introduces the principles of the system frame first. Then, it explains the basic principle of GMWM in Section 2.1 and presents the autonomous modeling in Section 2.2. The static state detection with adaptive threshold is developed in Section 2.3. Section 2.1–Section 2.3 detail the whole algorithm for online modeling of inertial errors. Section 3 details two conducted experiments to compare the algorithm proposed in this paper with other classical methods, and analyzes the positioning performance. Section 4 reveals the conclusions and further research directions.

## 2. Online Stochastic Error Modeling of Inertial Sensors

Generally, measurements of inertial sensors have various errors which contaminate the true measurements from the gyroscopes and the accelerators, decreasing the performance of the navigation solutions. For inertial sensors, the angular rate of the gyroscope and the specific force observation equation of the accelerometer are calculated as follows:

(1) $$\begin{array}{l} \omega =\omega_{true}+b_{\omega }+S_{\omega }\omega +c_{\omega }t+\varepsilon_{\omega }\\ f=f_{true}+b_{f}+S_{f}f+c_{f}t+\varepsilon_{f} \end{array}$$

where ω is the gyro output measurement, $\omega_{true}$ is the true rotation rate, $b_{\omega }$ is the gyroscope bias, $S_{\omega }$ is the gyroscope scale factor, $c_{\omega }$ is the gyroscope temperature coefficient, t is the temperature, f is the accelerometer output measurement, $f_{true}$ is the true specific force, $b_{f}$ is the accelerometer bias, $S_{f}$ is the accelerometer scale factor, $c_{f}$ is the accelerometer temperature coefficient, and $\varepsilon_{\omega }$ and $\varepsilon_{\omega }$ are the sensor noises. For IMU calibration, the six-position static calibration test is generally adopted to determine both the deterministic bias and scale factor of the gyros and accelerometers. Moreover, the temperature variation effect is neglected here in this paper due to the relatively short duration datasets. Thus, this research mainly explores the modeling and estimation of sensor noise, $\varepsilon_{\omega }$ and $\varepsilon_{f}$.

**Figure 1:**
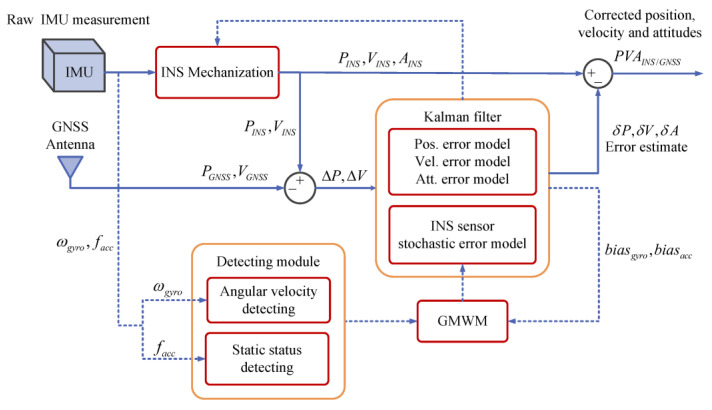
Layout of online stochastic error modeling of inertial sensors in INS/GNSS integration.

GMWM is a recently proposed technique to model the sensor noise $\varepsilon_{\omega }$ and $\varepsilon_{f}$. We make further improvements to GMWM to realize online modeling and design a novel navigation solution. It is designed within an INS/GNSS integrated system with feedback to provide information on IMU stochastic errors. The layout of the frame is shown in Figure 1. Here, we first introduce the basic principles of the system, then more details and some supporting conclusions will be further explained in Section 2.1–Section 2.3 of each module accordingly.

- When GNSS signal is received, the INS/GNSS integration within EFK obtains the navigation solution. Differing from traditional INS/GNSS integration, this system has an additional feedback to the EKF and has a stochastic error model as an augmented error vector in the EKF. When GNSS signal is blocked, the INS works in coasting mode;
- The feedback consists of autonomous stochastic error modeling of inertial sensors and the detecting module. The raw observations from the IMU will go to the detecting module. The static state detecting with adaptive threshold judges the motion state of the vehicle. If the vehicle is static, e.g., waiting before traffic lights or temporarily avoiding pedestrians or other vehicles, the IMU data accumulate during this static duration. After bias removal, accumulations will go to autonomous stochastic error modeling where the GMWM will prepare the best model of inertial stochastic error. Meanwhile, the angular rate detector will judge if the angular rate of the vehicle is higher than 30°/s. If not, the best model can work as an augmented vector together with the navigation error models in a Kalman filter. The Kalman filter outputs the bias of gyros and accelerators fed to autonomous stochastic error modeling in return;
- The IMU data for autonomous stochastic error modeling only accumulate during the static duration; therefore, the amount of data is quite small, and the calculation is generally completed within a few seconds. As the vehicle runs, the IMU data accumulation increases with extension of the static duration; consequently, the accuracy of the stochastic model improves.

### 2.1. Generalized Method of Wavelet Moments

GMWM is an estimation method based on the idea of generalized method of moments (GMM) estimators and the wavelet variance (WV) [16]. The GMWM makes use of the relationship between the WV and the parameters of a latent process, estimating the latter by minimizing the distance between the empirical WV and model-based WV [14]. The calculation process for the GMWM can be illustrated as follows:

The wavelet coefficients are built using wavelet filters $\left\{{\tilde{h}}_{j,l}:j=1,\cdots J\right\}$, where the j-th level wavelet filter of length is $L_{j}=(2^{j}-1)(L_{j}-1)+1$. In the stationary or non-stationary process, we get the maximum overlap discrete wavelet transform (MODWT) coefficients, $W_{j,k}$

(2) $${\bar{W}}_{j,k}=\sum_{l=0}^{L_{1}-1}{\tilde{h}}_{j,l}Y_{k-1},k\in Z$$

WV is defined as the variance of the wavelet coefficients, $W_{j,k}$, at the dyadic scales $\tau_{j}=2^{j-1}$.

(3) $$v^{2}(\tau_{j})=\mathrm{var}\left[{\bar{W}}_{j,k}\right]$$

For a finite observed process, the MODWT-estimated WV can be calculated as follows:

(4) $$v(\tau_{j})=\frac{1}{M_{j}}\sum_{k=L_{j}}^{N}{\bar{W}}_{j,k}^{2}$$

where $W_{j,k}^{}=\sum_{l=0}^{L_{j}-1}{\tilde{h}}_{j,l}y_{k-l},k\in (L_{j};N)$ and $M_{j}=N-L_{j}+1$. 

The PSD of the wavelet coefficient, $S_{W_{j}}(f)={\left|{\tilde{H}}_{j}(f)\right|}^{2}S_{F_{\theta }}(f)$, supports a direct relationship between WV and PSD, where the variance of the mentioned series of wavelet coefficients are the direct integral of its PSD as follows:

(5) $$v(\tau_{j})=\int_{-1/2}^{1/2}S_{W_{j}}(f)df=\int_{-1/2}^{1/2}{\left|{\tilde{H}}_{j}(f)\right|}^{2}S_{F_{\theta }}(f)df$$

where $H_{j}(f)$ is the transfer function of the filter $h_{j,l}$, $F_{\theta }$ is the model built using one or more stochastic processes that describes the dynamics of the observed sensor error sequence, and $S_{F_{\theta }}$ is the PSD implied by the model $F_{\theta }$. Therefore, there is an implicit connection between the WV and the parameters of the data generating model, $F_{\theta }$. We exploit this connection by defining an estimator for θ, namely by matching a sample estimate of the WV together with the model-based expression of the WV. The GMWM estimator is used to minimize the distance between the empirical and estimated WV in order to estimate the parameters of the latent composite processes as follows:

(6) $$\hat{\theta }=\underset{\theta \in \Theta }{\arg\min}{\left(\hat{v}-v(\theta )\right)}^{T}\Omega (\hat{v}-v(\theta ))$$

where θ represents the time series model parameter that we intend to estimate belonging to the compact set Θ, and Ω is a symmetric positive definite weighting matrix chosen in a suitable manner to make the GMWM estimator is as efficient as possible. It is also important to mention that this method could also be based on the AV since the aforementioned Haar WV is simply twice the AV with additional benefits. A detailed mathematical background on the GMWM can be found in [12,14].

The next step is the parameter estimation of the possible model, $F_{\theta }$, and ranking these models using specific criterion to determine the best one.

### 2.2. Autonomous Stochastic Error Modeling

#### 2.2.1. Complete Stochastic Error Model

Within our research interests, the problem of modeling and estimation focuses on the stochastic error components affecting gyroscopes and accelerators. Hence, we restricted the possible models, $F_{\theta }$, to a complex model which is defined as a combination of independent basic stochastic processes. These basic stochastic processes are widely used within the design of navigation filters and can precisely describe the behaviors of inertial sensors: gaussian white noise (WN), random walk (RW), drift (DR), quantization noise (QN) and finite auto regressive model (AR) [18,19,20,21,22,23].

In order to cover as many basic stochastic processes as possible, this paper defines the complete model of IMU stochastic error as

(7) error=4×AR+DR+WN+QN+RW

The complete model consisting of these basic stochastic processes is universally suitable to a variety of inertial sensors. When identifying the structure of the stochastic error, all the combinations of these basic stochastic processes within the complete model are regarded as the candidate models. Then, parameters of all the candidate models are estimated by GMWM and later, the best or most suitable model is selected by a designed model ranking criterion.

#### 2.2.2. Model Ranking Criterion

After using GMWM to estimate the parameters of all candidate models, it is necessary to establish a model ranking criterion according to the actual requirements of noise modeling and estimation in the practice appliance. According to this criterion, candidate models are evaluated and ranked to select the most suitable one. Ref. [15] gave a ranking criterion called wavelet variance information, which weighs the model fitness and computational complexity to evaluate the trade-off between the model accuracy and the estimation of the time-taken. Ref. [15] statically collected IMU data for several hours, leading to a significant amount of data. It makes the parameter estimation quite time-consuming, taking even up to several hours, especially when the model contains a large number of stochastic processes. However, for the online modeling problem of stochastic errors studied in this paper, a relatively small amount of IMU data are processed. Accordingly, the computational calculation is small, which leads to tiny difference between the time-taken for estimating different candidate models. Consequently, only the model accuracy is considered in this research here.

The objective function given by Equation (6) can be regarded as a mismatch between the WV calculated by the model $F_{\theta }$ and the WV calculated by observed measurements, and its purpose is to minimize this difference and make the model more closely match the observations. Based on this physical explanation, the ranking criterion can be defined as goodness of fitness (GOF) as follows:

(8) $$GOF={\left(\hat{\nu }-\nu (\hat{\theta })\right)}^{T}\Omega (\hat{\nu }-\nu (\hat{\theta }))$$

After the parameter estimation of all candidate models is completed by GMWM, all candidate models are evaluated by the GOF criterion, and the model with the smallest GOF value is selected as the optimal model.

**Figure 2:**
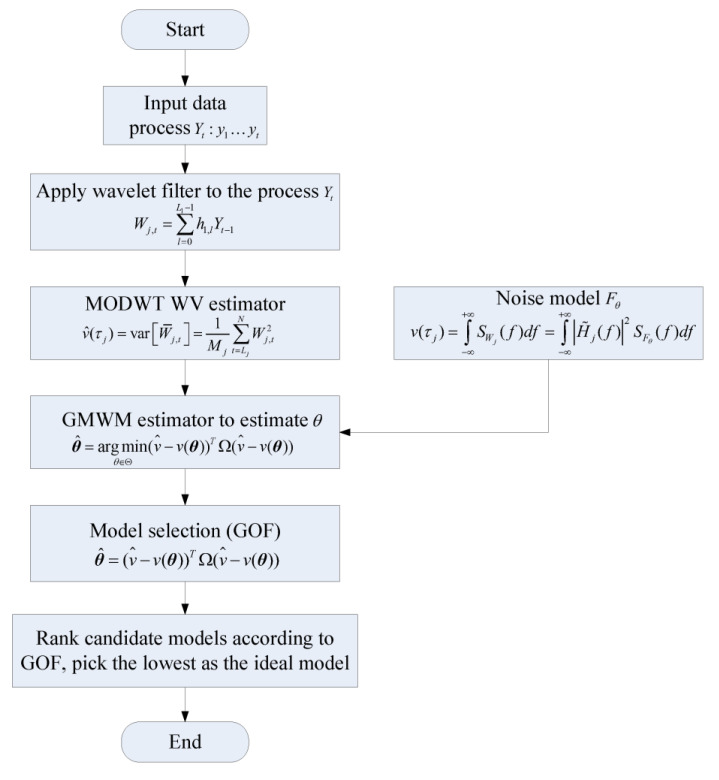
The overall flow of autonomous stochastic error modeling of inertial sensors.

A summary of the overall flow of autonomous stochastic error modeling of inertial sensors is shown in Figure 2.

### 2.3. Static State Detecting with Adaptive Threshold

For the vehicle navigation system, the inertial outputs contain some specific constraint information under different motion states. Under the premise of adding no extra cost and devices, the information can provide additional constraints for the navigation system, which is helpful to improve the accuracy and stability of the integrated navigation system. In particular, when the vehicle is in a static state, the inertial sensor is not affected by vehicle maneuvers, so the accelerations kept stable and the velocity remains close to zero. Nevertheless, the stability analysis of the accelerometer output can be performed to detect the static states. A common method is to use the standard deviation of the accelerometer output in a fixed time window as the test statistic [24]:

(9) $$\{\begin{array}{c} T_{i}(\;\mathrm{Accel}.\;X)<\lambda \\ T_{i}(\;\mathrm{Accel}.\;Y)<\lambda \end{array},T_{i}=\sqrt{\frac{1}{N-1}\sum_{j=i-N+1}^{i}{\left(A_{i}-U_{i}\right)}^{2}}$$

where $A_{i}$ is the accelerometer output at epoch i, $U_{i}$ is the mean value of the data in the fixed time window at epoch i, N is the number of data in the fixed time window, and N=100 in this paper. $T_{i}$ is the standard deviation of the data in the fixed time window at epoch i. Empirically, λ=0.02.

Due to the different characteristics of different inertial sensors, the standard deviation of the output in the static state is different as well. Hence, adopting a fixed empirical threshold is prone to misjudgment. Moreover, for the circumstances studied in this paper, it is crucial to ensure that the data for GMWM estimation modeling are from a static state. False detections have a more significant impact on the accuracy of modeling estimation than missed detections. Aiming at solving this problem, this paper proposes an adaptive method to determine the detecting threshold as follows:

(10) $$\lambda_{i}=\{\begin{array}{ll} 2\left(\frac{k-1}{k}\right)\lambda_{i-1}-\frac{\left|T_{i}-T_{i-1}\right|}{k} & ,T_{i-1}<\lambda_{i-1}\\ \lambda_{0} & ,T_{i-1}\geq \lambda_{i-1} \end{array}$$

where k is the number of the static states that has been detected at epoch i. $\lambda_{i}$ is the test statistic at epoch i, $\lambda_{0}$ is the initial value, and $\lambda_{{}_{0}}=0.02$, empirically.

The first term in Equation (10) physically means that when the standard deviation of the data is smaller than the test threshold, it is more inclined to assume that the vehicle stays in a continuous static state, so the detection threshold increases to make it easier to detect the static state. The second item physically means that if the standard deviation of the data between the current epoch and the previous epoch is quite large, the motion state of the vehicle has changed. It is more inclined to assume that the vehicle is not in the static state, so the detection threshold decreases to make it more difficult to detect the static state. The effect of this static state detection method with adaptive threshold will be experimentally verified in Section 4.

The static state detection with an adaptive threshold works together with the angular rate detection as the detecting module to provide extra guidance for autonomous stochastic error modeling. The detecting module is supported by two important conclusions given in [21]. One is that although stochastic errors do depend on the dynamic characteristics, for one specific IMU, the structure of the stochastic error is not affected by the applied dynamics. Only parameter values differ according to dynamic variations. The other is that for the general range of MEMS-IMU, the largest factor among various dynamic characteristics affecting stochastic errors is the angular rate. Moreover, a relatively low angular rate, normally below 30°/s, does not cause an evident change in the parameters. Fortunately, this is most commonly the case when turning in urban cities. Therefore, the angular rate detector will judge if the angular rate of the vehicle is higher than 30°/s. If not, the stochastic error model estimated under the static state can replace the dynamic model.

In practical applications, ranking all candidate models to identify the error structure will take up the most majority of the calculation time. However, identifying the structure will be conducted only once, i.e., after the first static duration, since the structure will not differ with the dynamic variations. Once the error structure is determined, only the parameter estimation of this fixed model structure will be conducted later through the experiment, and it can be processed within several seconds. It means that the autonomous stochastic error modeling lasts only a few seconds after the vehicle starts moving, then the KF will be able to adopt the stochastic error to obtain the navigation solution.

## 3. Experiments

This section is split into two main parts:

(1) GNSS denial experiments, which are designed to verify the feasibility of the autonomous stochastic error modeling based on GMWM;
(2) GNSS available experiments, which are designed to verify the feasibility of the proposed algorithm for online stochastic error modeling of inertial sensors. We compare the navigation performance of the proposed algorithm with the other traditional methods and further analyze the performance of the proposed algorithm in different trajectory sections as the car runs.

### 3.1. GNSS Denial Experiment

As previously mentioned, AV is probably the most commonly used method for model identification and sensor calibration. In 1998, the IEEE standard officially put forward this technique as a noise identification method to determine the characteristics of the underlying random processes that perturb data. In general, AV only considers five basic stochastic processes: QN, WN, BI, RW, and DR. These processes correspond to the linear regions in a log–log plot, which will present a typical U/V-shaped curve in ideal circumstances. Therefore, parameters are usually estimated by performing linear regression of (visually) identified linear regions in such log–log plots. Further research on this principle can be found in [8,9,10,25,26]. A GMWM-based algorithm can be considered as a further extension of AV because they both identify and quantify the different noise terms that exist in inertial sensor data. Hence, in this experiment, we compare the three models with different GOF values generated by the autonomous stochastic error modeling method, AV method, and loosely coupled navigation solution with a reference trajectory.

**Figure 3:**
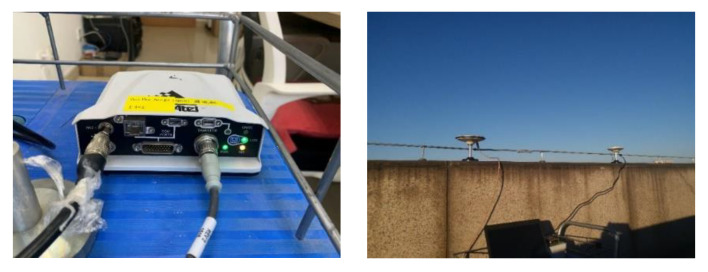
SPAN NovAtel (**left column**) is placed statically indoors and the antenna (**right column**) is set up outdoors to collect GNSS signals.

**Figure 4:**
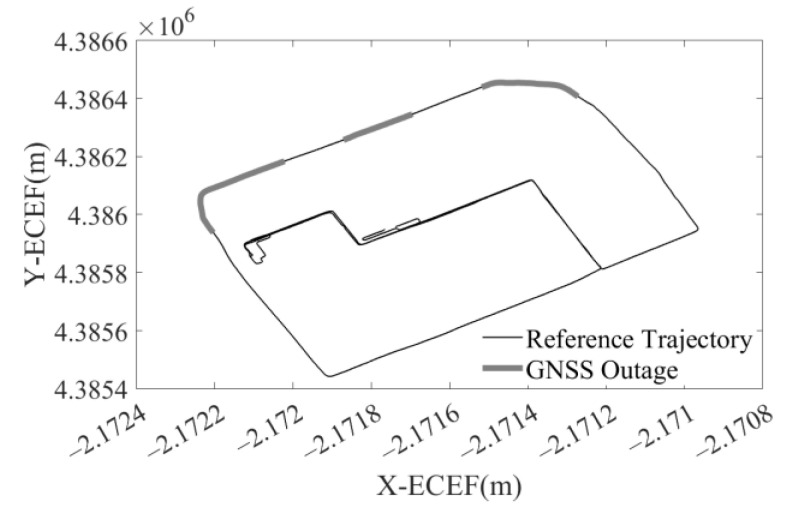
The reference trajectory and three artificial GNSS outages.

The trajectory was produced by a car with SPAN NovAtel-CPT driving around an urban area of Beijing on 18 June 2021. The reference is provided by SPAN NovAtel-CPT under the post-processed solution. The raw IMU data were collected statically for 2 h at 125 Hz by SPAN NovAtel-CPT, as shown in Figure 3. After the data were fed to the autonomous stochastic error modeling method, we adopted three models with the minimum GOF values to analyze the performance as an example. Three artificial GNSS outages, each lasting 60 s, were designed to cover two turns and one straight line, as shown in Figure 4. The planimetric navigation drift in the earth centered earth fixed (ECEF) coordinate system at the end of GNSS outages was analyzed to judge the quality of the five different models.

#### 3.1.1. Stochastic Error Modeling

**Figure 5:**
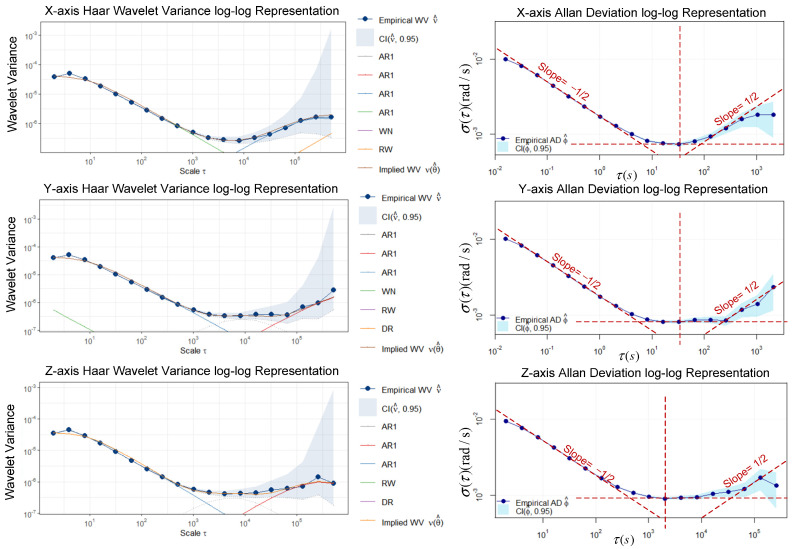
GMWM log–log plot of the three-axis gyroscope (**left column**) and AV log–log plot of the three-axis gyroscope (**right column**). The red dashed lines are auxiliary lines to help identify linear regions.

Based on the optimal model autonomous selection method proposed in Section 3.2, the 2 h static IMU data after bias removal were processed by the GMWM-based method and the AV-based method. A GMWM log–log plot of the best model with the minimum GOF and an AV log–log plot is shown in Figure 5. It implies that the GMWM-based method proposed in this paper can achieve a good fit to the static data. The curves in the AV log–log plot represent the characteristics of three stochastic processes, i.e., WN, BI, and RW. The WN parameters can be deduced from a slope of −1/2 at τ=1 on the left part. BI due to flicker noise in the measurements can be identified at the lowest point in the curve. The RW parameters can be deduced from a slope of 1/2 on the right part.

#### 3.1.2. Experiment Validation

The five gyro stochastic models to be validated are as follows:

Model 1: Loosely coupled navigation solution;

Model 2: AV-based model;

Model 3–5: GMWM-based model with three minimum GOF values (values decrease from 3 to 5).

**Figure 6:**
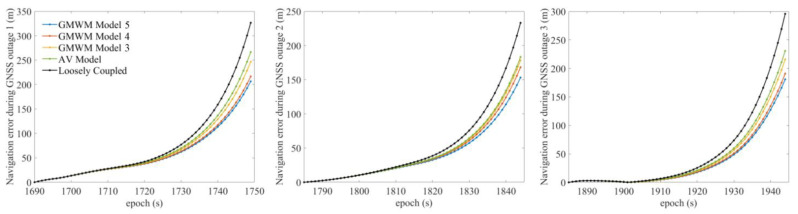
The navigation drift of the five models at the end of three GNSS outages.

The navigation drifts of the five models at the end of three GNSS outages are shown in Figure 6, and the maximum navigation errors of the five models at the end of three GNSS outages are shown in Table 1. Figure 6 indicates that during the three GNSS outages, the GMWM-based model generally outperforms the other two models, with the AV-based model in the middle of the two models. Among the three models generated by the GMWM method, the best model with minimum GOF values gives the best results with 206 m, 153 m, and 182 m during three outages, respectively. Compared with the loosely coupled navigation solution, the best GMWM model has an accuracy increase of 26.8%, 37.2%, and 38.3% during three outages, respectively.

### 3.2. GNSS Available Experiment

As mentioned above, human judgment in the identification of five noise terms will absolutely cause deviation in estimation parameters when performing Allan variance. Aiming to solve this problem, reference [27] provided a method to automate this process by maximizing the likelihood function of the assumed state-space models of interest using a constrained version of the expectation maximization (EM) algorithm [28]. Hence, we compared the algorithm for online stochastic error modeling proposed with the AV-based method, the EM-based method, and the traditional EKF solution to verify its efficiency.

**Figure 7:**
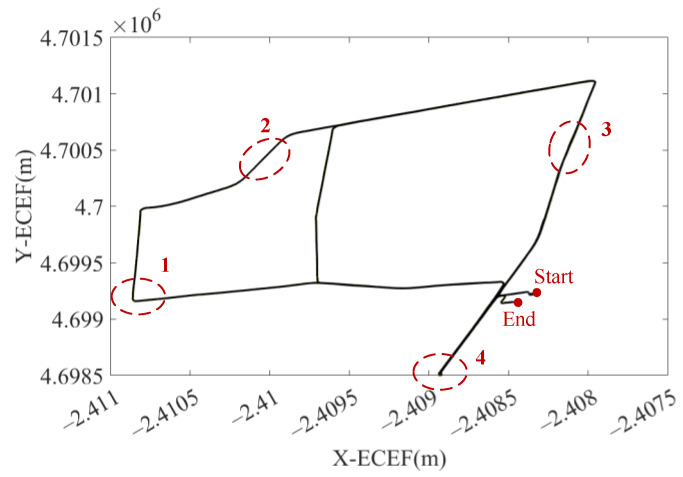
The reference trajectory of the GNSS available experiment in ECEF. The end and the start is marked in red point. Four sections are given detailed figures for further analysis (in dashed red circle).

In this experiment, the trajectory was produced by a car with SPAN NovAtel-CPT driving around an urban area of Xuzhou on 16 April 2018. The reference trajectory was provided by SPAN NovAtel-CPT under the post-processed solution, as shown in Figure 7. The whole trajectory lasts 50 min and the IMU data were sampled at 125 Hz. The IMU data accumulate during the static epochs detected by static state detecting with an adaptive threshold. Autonomous modeling prepares the best stochastic error model as the augmented vector in INS/GNSS integration with EKF.

#### 3.2.1. Static State Detection

**Figure 8:**
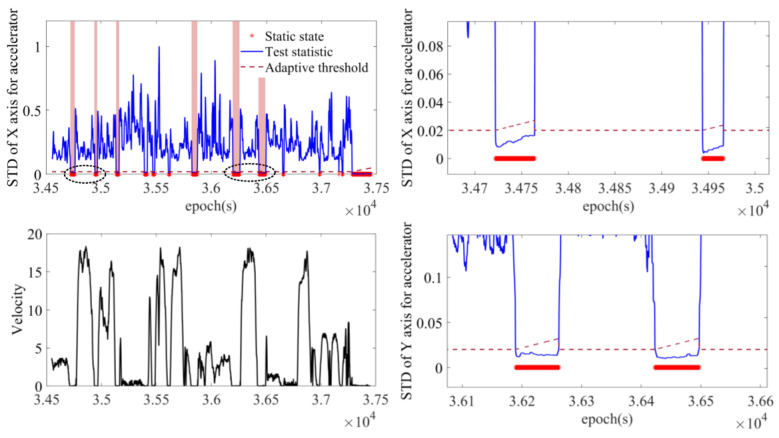
The result of static state detection with adaptive threshold compared with the velocity curve (**left column**) and two detailed figures of static states (**right column**). Two sections are given detailed figures for further analysis (in dashed black circle).

The result of static state detection with adaptive threshold for the X axis during the whole trajectory is shown in Figure 8. Comparing the curve of test statistic and car velocity, it is clear that this method successfully detects almost all of the static states, including six relatively long stops and other temporary stops. The six long stops are marked by the light red area for further experimental analysis. For the static states marked by the red stars, the standard deviation statistics of the fixed window have obvious statistical characteristics, i.e., they blow the adaptive threshold, which can effectively identify the static epochs. In particular, in the right column are two zoomed-in figures of the detected results. It shows how the threshold (dark red lines) adjust to the actual circumstances of the detection. Since the previous epochs are identified as static states, the adaptive threshold increases to extend this inertia. However, when the test statistic fluctuates sharply, the adaptive threshold shows an immediate decrease.

#### 3.2.2. Stochastic Error Modeling

**Figure 9:**
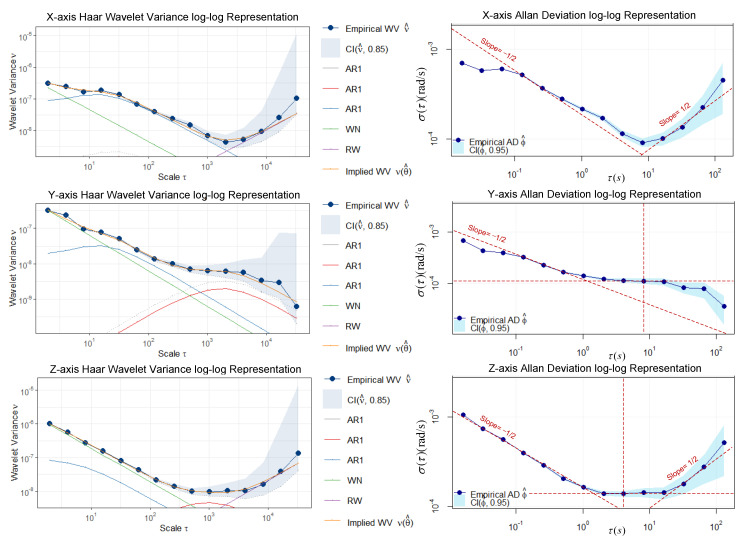
A GMWM log–log plot of the three-axis gyroscope (**left column**) and an AV log–log plot of the three-axis gyroscope (**right column**). The red dashed lines are auxiliary lines to help identify linear regions.

Based on the detection results of static states, raw IMU data accumulate during the static epochs, reaching a duration of 318 s. The accumulation of static data was fed to the GMWM-based model, the AV-based model, and the EM-based model. For a clear view of the estimation results of these two methods, Figure 9 simply gives the estimation results based on all the static data accumulated throughout the whole trajectory. The parameter estimation results of the AV-based method, the EM-based method, and the GMWM-based method are shown in Table 2, Table 3 and Table 4, respectively.

It is worth mentioning that AV plot-plot of X axis and Y axis does not present a typical U- or V-shaped curve. Only WN and RW can be identified from the X axis and only WN and BI can be identified from the Y axis. The amount of data accumulation is quite small; therefore, it requires a much larger amount of data to present the specific characteristics of other processes. Moreover, the human identification in each process is probably unreliable, then the parameters estimated by performing linear regression of these visually identified linear regions may have significant deviations. The EM-based approach is very sensitive to the initial values of parameters. When the initial values are “far” from the true values, the EM-based approach is likely to converge to a local minima. Hence, the initial values are set to the results estimated by AV.

#### 3.2.3. Experiment Validation

Based on the stochastic error model generated above, three models were compared as follows:

Model 1: INS/GNSS integration within EKF;

Model 2: EKF with AV-based model;

Model 3: EKF with EM based model;

Model 4: EKF with an online GMWM-based model.

**Figure 10:**
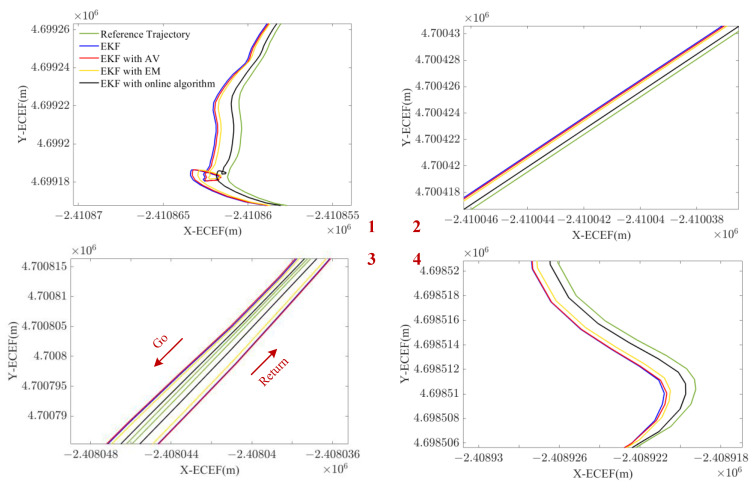
Four detailed figures of trajectories generated by the four models.

**Figure 11:**
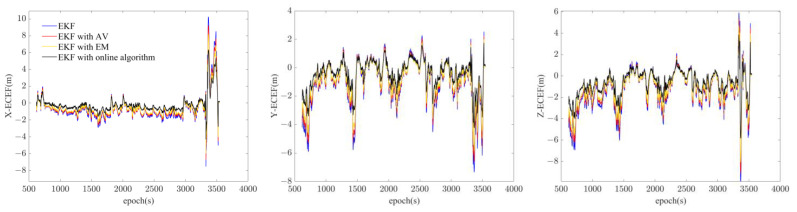
The navigation error of the three models in X, Y, and Z of ECEF.

The reference trajectory is shown in the left column in Figure 10 and the four detailed figures of trajectories generated by the four models are shown on the right. It is clear that the algorithm proposed is the closest to the reference trajectory regarding both turns and straight lines. Figure 11 indicates the navigation error of the three models in X, Y, and Z of the ECEF coordinate system. The online GMWM model reflects a more accurate positioning result than the other two models. Table 5 shows the navigation error root mean square error (RMS) of the three models. It clarifies that the online GMWM model has the smallest navigation errors of 1.3265 m, 1.4384 m, and 1.6629 m in X, Y, and Z, respectively. Compared with the AV-based method, it has an improvement of 16.6%, 14.3%, and 14.7% in the navigation accuracy of X, Y, and Z, respectively. Compared with the EM-based method, it has an improvement of 10.8%, 8.2%, and 7.1% in the navigation accuracy of X, Y, and Z, respectively. It significantly validates the effect of the online algorithm.

Moreover, the stochastic error model become more and more accurate with the accumulation of the static data. Table 6 shows the navigation error RMS of the online algorithm for the trajectory sections between two adjacent long stops. The positioning accuracy increases in X, Y, and Y as the car drives, which further confirms the ability of the online GMWM algorithm to enhance the navigation accuracy.

Particular attention should be given to the time-taken for the online algorithm. All the static epochs detected take up 318 s in the whole trajectory. Due to the relatively small amount of data accumulation, calculations with the online algorithm take little time. After the first relatively long stop, identifying the model structure and estimating the parameters takes 1.4 min. After that, only the estimation of the fixed model structure will be performed, which takes much less time, i.e., it is finished within 3 s. Table 6 also gives the time-taken after six long stops in the right column. After the sixth long stop, the static data accumulated reach the largest amount in the whole trajectory. It means that the stochastic error model can work as an augmented vector within EKF solution, with a delay of 2.434 s. Therefore, the algorithm has an excellent performance in online modeling of stochastic errors.

## 4. Conclusions

This paper proposes a new method for online modeling of stochastic errors of inertial sensors, which combines static state detection with an adaptive threshold and the autonomous stochastic error model based on GMWM. Firstly, the limitations of other widely used stochastic modeling methods including AV and PSD are analyzed. Then, two experiments are designed to compare the online GMWM algorithm with the AV-based method and the EM-based method. The GNSS denial experiment proves the feasibility of the proposed autonomous stochastic error model based on GMWM. Meanwhile, it reveals the capability of the online GMWM algorithm to estimate the stochastic error and limit the navigation drift. The GNSS available experiment demonstrates that the online GMWM can significantly improve the navigation accuracy as the vehicle runs. Moreover, it validates the online performance as well.

This paper mainly focuses on exploring the stochastic error of inertial sensors, which is related to the natural characteristics of the sensor itself. Meanwhile, for vehicle-mounted sensors, the dynamic environment of the vehicle may affect the error behavior of the sensor as well. Given the conclusions of our research, a stochastic error model in a static environment can work instead of a model at low speed. Therefore, further study is warranted on how to construct the error models and estimate the parameters based on dynamic characteristics. We will focus on how the inertial error model changes with vehicle maneuvers at high speed. Future work aims to set up and conduct experiments which enable the construction and analysis of error signals acquired in dynamic environments. Therefore, the observability of some processes and the justification of employing complex stochastic models for MEMS inertial sensors can only then be fully verified.

## Figures and Tables

**Table 1:** The maximum navigation error of the five models at the end of three GNSS outages.

Model		Navigation Drift at the End of GNSS Outage (m)		
		Outage 1	Outage 2	Outage 3
Loosely coupled		326	243	295
AV		264	186	227
GMWM	Model 3	254	178	215
	Model 4	216	164	191
	Model 5	206	153	182

**Table 2:** The parameter estimation results of AV-based method.

Parameter	X	Y	Z
$\sigma_{WN}$	$1.763\times 10^{-3}$	$1.347\times 10^{-2}$	$1.309\times 10^{-2}$
$\sigma_{RW}$	$7.171\times 10^{-3}$		$6.334\times 10^{-3}$
$\sigma_{BI}$		$1.762\times 10^{-4}$	$1.407\times 10^{-4}$
$T_{BI}$		4.096	4.096

**Table 3:** The parameter estimation results of EM-based method.

Parameter	X	Y	Z
$\sigma_{WN}$	$1.822\times 10^{-3}$	$1.441\times 10^{-2}$	$1.358\times 10^{-2}$
$\sigma_{RW}$	$7.167\times 10^{-3}$		$6.401\times 10^{-3}$
$\sigma_{BI}$		$1.759\times 10^{-4}$	$1.413\times 10^{-4}$
$T_{BI}$		4.092	4.093

**Table 4:** The parameter estimation results of GMWM-based method.

Parameter		X	Y	Z
		Model = 3 × AR + WN + RW	Model = 3 × AR + WN + RW	Model = 3 × AR + WN + RW
AR	$\sigma_{1}$	$3.009\times 10^{-9}$	$7.156\times 10^{-11}$	$4.461\times 10^{-10}$
	$\beta_{1}$	$8.574\times 10^{-1}$	$9.979\times 10^{-1}$	$9.298\times 10^{-1}$
	$\sigma_{2}$	$1.723\times 10^{-9}$	$4.498\times 10^{-11}$	$1.871\times 10^{-10}$
	$\beta_{2}$	$8.852\times 10^{-1}$	$9.978\times 10^{-1}$	$9.961\times 10^{-1}$
	$\sigma_{3}$	$3.064\times 10^{-7}$	$6.96\times 10^{-8}$	$2.340\times 10^{-7}$
	$\beta_{3}$	$7.512\times 10^{-1}$	$7.611\times 10^{-1}$	$3.866\times 10^{-1}$
WN	$\sigma_{WN}$	$4.551\times 10^{-7}$	$6.264\times 10^{-7}$	$1.878\times 10^{-6}$
RW	$\sigma_{RW}$	$1.276\times 10^{-11}$	$4.171\times 10^{-15}$	$2.506\times 10^{-11}$

**Table 5:** The navigation error RMS of the three models.

Model	Navigation Error RMS (m)		
	X	Y	Z
Model 1	1.6675	1.7170	2.0956
Model 2	1.5916	1.6802	1.9492
Model 3	1.4877	1.5665	1.7902
Model 4	1.3265	1.4384	1.6629

**Table 6:** The navigation error RMS of the online GMWM algorithm between the two adjacent long stops and the time-taken after long stops.

Stops	Navigation Error RMS (m)			Time-Taken
	X	Y	Z	
1–2	1.5379	1.6442	1.8184	1.4 min
2–3	1.5267	1.6279	1.7950	1.877 s
3–4	1.4860	1.6118	1.7692	1.996 s
4–5	1.4369	1.5543	1.6974	2.184 s
5–6	1.3872	1.4265	1.6483	2.338 s
6–end	1.2969	1.4098	1.6318	2.434 s

## Data Availability

Not applicable.
